# Combined alignments of sequences and domains characterize unknown proteins with remotely related protein search PSISearch2D

**DOI:** 10.1093/database/baz092

**Published:** 2019-07-17

**Authors:** Minglei Yang, Wenliang Zhang, Guocai Yao, Haiyue Zhang, Weizhong Li

**Affiliations:** 1 Zhongshan School of Medicine, Sun Yat-sen University, Guangzhou, China; 2 Center for Precision Medicine, Sun Yat-sen University, Guangzhou, China; 3 Key Laboratory of Tropical Disease Control (Sun Yat-Sen University), Ministry of Education

## Abstract

Iterative homology search has been widely used in identification of remotely related proteins. Our previous study has found that the query-seeded sequence iterative search can reduce homologous over-extension errors and greatly improve selectivity. However, iterative homology search remains challenging in protein functional prediction. More sensitive scoring models are highly needed to improve the predictive performance of the alignment methods, and alignment annotation with better visualization has also become imperative for result interpretation. Here we report an open-source application PSISearch2D that runs query-seeded iterative sequence search for remotely related protein detection. PSISearch2D retrieves domain annotation from Pfam, UniProtKB, CDD and PROSITE for resulting hits and demonstrates combined domain and sequence alignments in novel visualizations. A scoring model called C-value is newly defined to re-order hits with consideration of the combination of sequence and domain alignments. The benchmarking on the use of C-value indicates that PSISearch2D outperforms the original PSISearch2 tool in terms of both accuracy and specificity. PSISearch2D improves the characterization of unknown proteins in remote protein detection. Our evaluation tests show that PSISearch2D has provided annotation for 77 695 of 139 503 unknown bacteria proteins and 140 751 of 352 757 unknown virus proteins in UniProtKB, about 2.3-fold and 1.8-fold more characterization than the original PSISearch2, respectively. Together with advanced features of auto-iteration mode to handle large-scale data and optional programs for global and local sequence alignments, PSISearch2D enhances remotely related protein search.

## Background

Remotely related protein search refers to detection or identification of homologous proteins, which are similar in structure and function but with low identity in sequence level (1). It is one of the fundamental techniques for gene annotation, protein structure classification and function prediction. With the development of high-throughput sequencing techniques, over 45 million protein sequences (more than 36% of all) remain unknown or uncharacterized in UniProtKB (Release 2018_08 12-Sep-2018) (2). However, detecting the remote homologs with low sequence similarity is much harder than detecting the close homologs with high similarity. It has become an emergent task to develop effective computational approaches to tackle the challenges in the identification of remotely related proteins.

Iterative homology search has been widely used in identification of remotely related proteins. Sequence-based iterative methods such as PSI-BLAST (3, 4) and profile-based iterative methods such as HHblits (5) and jackHMMER (6, 7) can be 5–100-fold more sensitive than single sequence search (8, 9), but unfortunately result in unrelated homologs due to contaminated position-specific scoring matrices (PSSMs) (10), mainly caused by homologous over-extension (HOE) errors (11). Several years ago, we reported a sequence masking method (PSISearch) (12) that can reduce HOEs in interactive homologous search and gain 4–5-fold better selectivity for homologs. Recently, we have found that a query-seeded sequence iterative search method (PSISearch2) (13) can remove more HOEs and improve selectivity about 20-fold compared with PSI-BLAST and jackHMMER, with little loss in sensitivity. However, iterative homology search remains challenging in protein functional prediction and genomic annotation. For example, many proteins identified in large-scale screens remain uncharacterized, but sequence alignment methods usually offer low-confident homologs in characterizing unidentified proteins. Moreover, resulting hits are difficult to interpret and classify when E-values of hits are close to the E-value threshold. More sensitive scoring models are highly needed to improve the predictive performance of the alignment methods, and alignment annotation with better visualization has also become imperative for result interpretation.

Protein domain detection has been used to identify related proteins as well (14). Several databases, such as Pfam (15), InterPro (16), CDD (17), CATH (18), Gene3D (19) and PROSITE (20), annotate proteins with domains and other protein signatures, as well as providing search tools to detect domains for unknown proteins. Manual curation of combined information from sequence alignments and domain annotation can result in highly confident annotation for uncharacterized proteins. However, currently no tools are available to combine domain alignment with sequence alignment to confidently identify the relationship of related proteins.

Here we report an open-source application PSISearch2D that runs query-seeded iterative sequence search against protein sequence data for remotely related protein detection. PSISearch2D retrieves domain annotation from Pfam (15), UniProtKB (2), CDD (17) and PROSITE (20) for resulting hits and demonstrates combined domain and sequence alignments in novel visualizations to ease result interpretation. A scoring model called C-value is newly defined to re-order hits with consideration of the combination of sequence and domain alignments. PSISearch2D improves the predictive performance by combining alignments of sequences and domains and is able to improve characterization of unknown proteins in remote protein detection. Our evaluation tests show that PSISearch2D characterizes more unknown proteins than the original PSISearch2 tool (13). Moreover, PSISearch2D provides auto-iteration mode to handle large-scale input and optional programs of GGSEARCH/GLSEARCH/PSI-BLAST for global and local sequence alignments.

## Methods

### Iterative sequence search

**Figure 1:**
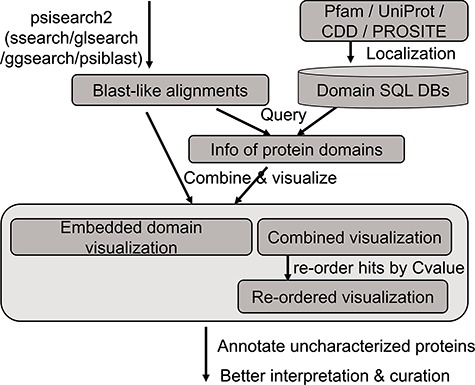
The analysis flow chart of domain annotation and visualization in PSISearch2D.

An updated query-seeded method of PSISearch (13) is implemented in this PSISearch2D (Figure 1). It processes query-seeded search in each iterative run against protein sequence databases, produces BLAST-like output alignments and retrieves Pfam, UniProtKB, PROSITE or CDD domain annotation for query and subject sequences. In homologous sequence search, query represents the query sequence during the search submission and subjects represent the hits in the search result. To offer convenience for remotely homologous searches against specified species, the subject databases include UniProtKB (SwissProt + TrEMBL) and its database subgroups based on model species and taxonomy categories. The database subgroups contain protein sequences of bacterial, virus, archaea, eukaryotes and model species including human, mouse, rat, zebrafish and *E. coli*. The subject databases are updated every 6 months. PSISearch2D implements SSEARCH (21) as default search program for Smith–Waterman local alignment (22) and also provides other advanced search programmes such as GGSEARCH-/GLSEARCH-/PSI-BLAST-based iterative search as extra options for global-global, global-local (21) or psi-blast sequence alignments, respectively.

### Database construction of domain annotation

The annotation data of protein domains were retrieved from the databases of UniProtKB (Swiss-Prot and TrEMBL, Release 2019_01), Pfam (Version 32.0), PROSITE (Release 2019_02) and CDD (Release 2018_05) (Figure 1). Considering the heterogeneous domain annotations in these databases, only protein domains marked with key annotation of ‘domain’ and ‘region’ were extracted from the downloaded flat files by in-house pipeline and stored in tab-delimited flat files. The extracted domain information includes the entries’ accession numbers, start and end positions of domains, domain names and descriptions. These information in the flat files were then imported into a local MySQL database for further indexing and searching.

### Querying domain annotation

A high-speed retrieval index for each table was created for domain annotation in MySQL. A Perl script wrapped in the PSISearch2D uses the Perl DBI module to query the high-speed indices. It also associates both query and subject proteins with domain annotation information including names and boundary coordinate based on the protein accession numbers (Figure 1). The resulting domain annotations are sorted according to the domain position on the sequence. The localization of domain databases avoids large-scale remote requests of Pfam and UniProt web service through the Internet and thus accelerates the query processes of domain annotation.

### Visualizing domain alignments

**Figure 2:**
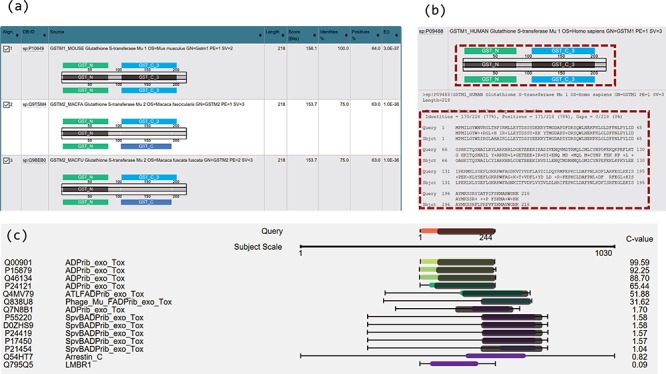
Novel visualizations for domain and sequence alignments. (a) Result summary table shows the domain alignments. (b) Domain alignment and sequence alignment in the same window allow comparison of the two types of alignments and ease the interpretation for the resulting hits. (c) Sequence and domain alignments are combinedly displayed in the view of multiple hits.

Two novel approaches of visualization have been implemented by using JavaScript to improve result interpretation. Firstly, embedded domain alignments are displayed with sequence alignments in result summary tables. For example, Figure 2a displays two domains in the query GSTM1_MOUSE protein (UniProt accession number P10649) and most of the top hits contain the same or similar domains. In the view of embedded domain alignment, the sequence alignment between the query and subject sequences is shown as a black-line box in the middle part of the view. The black lines cross this box represent the query and the subject sequences, respectively. The colour boxes over the middle black-line box represent the domains of the query along with the sequence alignment, and those under the middle black-line box stand for the domains of the subject. The grey boxes inside the middle black-line box represent the matched domains between the query and the subject. The grey box does not exist if no matched domains are between them. Domain names are labelled in the domain boxes. By expanding the sequence alignments in the summary table, users can easily compare the domain alignments with the sequence alignments in the same page (Figure 2b). This visualization allows confident identification of true or false positives based on both sequence alignments and domain alignments, and at the same time enables better selection of hits for further analysis.

Secondly, sequence and domain alignments are combinedly displayed in the view of multiple hits. For example, in Figure 2c, the coloured bars in the middle column represent the sequence alignments. The red bar in the top part in the figure represents the query sequence and the bars in other colours represent the hits. The hits are coloured according to E-values/C-values of the hits. The lengths of the coloured bars stand for the lengths of the sequence alignment regions. The grey bars along with the bars of hits illustrate the matched domains over the hits, and their lengths represent the lengths of the domains. Figure 2c clearly illustrates the patterns of the sequence and domain alignments for multiple hits in one visualization. Users can navigate both the sequence alignments and the domain alignments across multiple hits.

### C-value to re-order hits close to E-value threshold

A newly defined score C-value is computed to measure significant levels of combination of sequence and matched domain alignments as well as to re-order the resulting hits. It is designed to assess the hits that have E-values close to threshold. It is computed by considering both E-value and the matched domains of subject sequences. Please see the equations as below.

(1) \begin{equation*} {w}_d=-{\log}_{10} eValueThreshold \end{equation*}

(2) \begin{equation*} cValue={w}_d\ast \frac{\sum_{j=0}{matchDomainLength}_j}{subjectLength}-{\log}_{10} eValue \end{equation*}

In Equation (1), *eValueThreshold* is the E-value cut-off chosen by users in search submission (<1), and *W_d_* is the weight for domain alignments based on the chosen E-value threshold. In Equation (2), *cValue* refers to the final score C-value, *eValue* is the E-value of pair-wise sequence alignment, *matchDomainLength*_j_ represents the length of matched domain *j* in the subject sequence and *subjectLength* stands for the length of subject sequence. Matched domains of subjects refer to the domains that are the same as the domains in the query. If a query sequence is not directly associated with a protein domain, the domains of the top hit will be treated as the query’s domains in the C-value calculation and domain alignment visualization.

The impact of domains in the calculation of C-value depends on how long the matched domains are aligned on the subject sequence and the weight of domain (*W_d_*) according to the chosen E-value threshold. When assessing hits with E-value close to threshold, the weight of domain (*W_d_*) roughly equates to the impact of E-value. Usually, E-value threshold for iterative homolog search is set as e-3 to e-5. C-value threshold can be set as 4 for low stringency and 6 for high stringency. C-value as 4 roughly equates to the stringency of E-value e-3 with matched domains aligning onto one-third of the full length of subject sequence; C-value as 6 roughly equates to the stringency of E-value e-3 with matched domains aligning onto the full length of subject sequence.

### Web application implementation

The standalone version of PSISearch2D implements the above steps in an auto-iteration mode to facilitate high-throughput remote protein search, costing much less time than the manual-iteration mode. Moreover, the PSISearch2D web application (http://lilab2.sysu.edu.cn/psisearch2d) was implemented with the JDispatcher technology (23) that is a Spring MVCbased tool framework for bioinformatics sequence analysis. Tomcat was used as the application container and JavaScript was used for data visualization.

**Figure 3:**
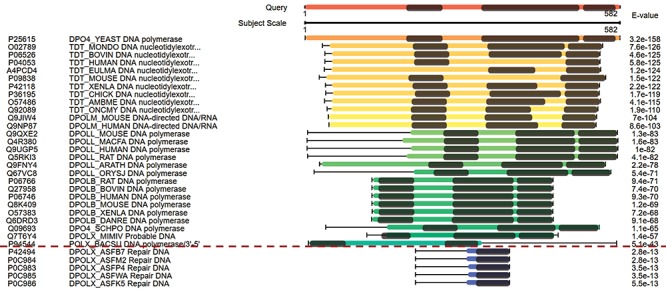
The combined domain and sequence alignments in the view of multiple hits easily excludes false-positive hits. The first column from the left side refers to the accession numbers of hits, the second column refers to the protein descriptions, the third column virtualizes the alignments and the last column indicates the E-values of the hits.

## Results

### Visualization of combined alignments

**Figure 4:**
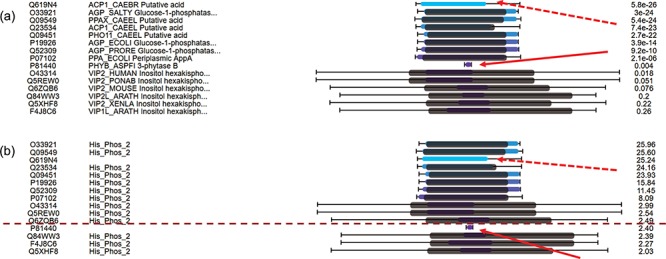
C-value to re-order hits for easy classification of true/false hits. (a) Domain alignments along with sequence alignments in the view of multiple hits ordered by E-values. The first column from the left side refers to the accession numbers of hits, the second column refers to the protein descriptions, the third column virtualizes the alignments and the last column indicates the E-values of the hits. (b) Domain alignments along with sequence alignments in the view of multiple hits re-ordered by C-values. The first column from the left side refers to the accession numbers of hits, the second column refers to the protein domains, the third column virtualizes the alignments and the last column indicates the C-values of the hits.

The PSISearch2D application displays embedded domain alignments in a summary table (Figure 2a) and re-orders homologous hits in visual outputs of combined alignments (Figure 4) according to C-values. Users can interpret results easily with both domain and sequence alignments and make better selection of hits for further analysis. Figure 2a is an example of search of GSTM1_MOUSE protein (UniProtKB accession P10649) against UniProtKB with Pfam domain annotation and visualization, showing the domain alignment for each hit along with the hit description. The visualization of domain alignments demonstrates clearly whether the query protein and the subject protein have the matched or mismatched domains embedded in the sequences. In this example, the query has domain GST_N and GST_C, and the fourth hit (GSTM1_HUMAN) has the same domains; moreover, the domains embed in the matched positions in the query and the fourth hit: GST_N on the left side and GST_C on the right. These imply the fourth hit is a perfect hit of the query, better than second and third hits. Furthermore, the result summary table allows comparison of domain alignment and sequence alignment in the same window (Figure 2b). This eases the interpretation of the results.

In general, sequence alignment visualization only displays sequence alignments (24). In some cases, proteins of different functions have similar sequence structures. Their sequence-level alignments are similar but the domain alignments could be different. To tackle this problem, PSISearch2D displays the domains along with sequence alignments in the view of multiple hits. Figure 2c shows the domains in the grey bars over the sequence alignment and allow users to easily check if the hits have matched domains. For example, Figure 2c shows the multiple hits for a search of ARC3_CBCP (Mono-ADP-ribosyltransferase C3 in *Clostridium botulinum* C phage) against UniProtKB_Swissprot. This remote homolog search resulted in 14 hits. The top six hits have matched domain alignments over the sequence alignment with high C-values (low E-values), which indicates they are true-positive hits.

Figure 3 displays the domain alignments along with sequence alignments in the view of multiple hits. The query sequence on the top of Figure 3 contains three domains; therefore, three domains are expected in the true-positive hits. Although the hits under the red dash line in Figure 3 have good E-values, they are short in length and contain only one domain. These suggest that they are false-positive hits. This view of multiple hits helps to easily exclude false-positive hits from the hits with good E-values.

### Re-order hits by C-value

For hits around E-value thresholds (usually e-3), users usually find it difficult to decide whether they are true or false hits. To address this issue, C-values are used to re-order the hits. Figure 4a displays the multiple hits of query MINP1_CHICK (multiple inositol polyphosphate phosphatase, accession F1NPQ2). P81440 has a very short sequence alignment with the query, and this implies that it is a false hit (indicated by red solid arrows). However, it is ranked higher than other true positive hits according to the E-values. By using C-value to re-order the hits, P81440 is moved down to the lower ranking area (Figure 4b) to indicate its less significant level. Also, the hit Q619N4 has a long sequence alignment with query, but no matched domain. This suggests extra attention when interpreting Q619N4. By using C-value, Q619N4 is ranked down as well (indicated by red dashed arrows in Figure 4).

**Figure 5:**
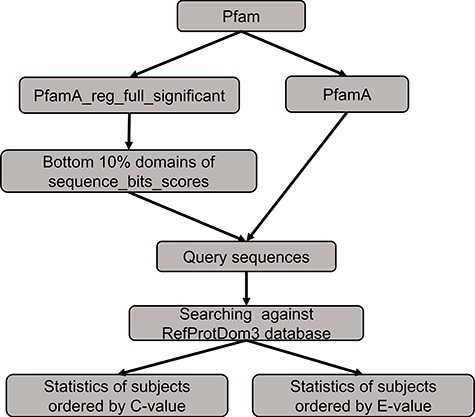
The benchmarking method to compare the performance between PSISearch2D and the original PSISearch2.

### Benchmarking on the use of C-value

We conducted a benchmarking to compare the performance between PSISearch2D and the original PSISearch2 tool (13). The method of the benchmarking is illustrated in Figure 5. We had to build a random set of query sequences before conducting searches for comparison. Firstly, the pfamA_reg_full_significant table and the pfamA table were downloaded from Pfam28 (15). The former table contains information from the Pfam domain alignments and the latter one includes the domain information such as domain lengths. The pfamA_reg_full_significant table was sorted by the sequence bits scores in the table, and the sorted domains of the bottom 10% were selected. Then, according to the domain lengths from the pfamA table and the domains alignments from the pfamA_reg_full_significant table, we randomly selected 100 query sequences that have sequence length over 50 amino acids and domain regions covering at least 50% of the domain length from the selected domains. Finally, these query sequences were searched against the RefProtDom3 database (25) by PSISearch2D and PSISearch2. The information of the query sequences and the result summary of the searches can be found in the supplementary file (http://lilab2.sysu.edu.cn/psisearch2d/suppl/benchmarking_result.xlsx).

We collected the information of top five hits and hits better than thresholds to compare the performance between PSISearch2D and the original PSISearch2. Following the same approach adopted in our previous study (13), we defined that true positive hits contain matched domains to query sequences and false positive hits contain different domains to the queries. Table 1 shows the summary results of the benchmarking. Firstly, the top five hits sorted by C-value from PSISearch2D and the top five hits sorted by E-value from PSISearch2 were included for the comparison. Of the total 368 top five hits, the number of true positive hits (330) by PSISearch2D is about 5% larger than that (311) by PSISearch2, and the number of false positives (38) by PSISearch2D is about 33% fewer than that (57) by PSISearch2. Secondly, the hits over the thresholds with C-values larger than six from PSISearch2D were compared with the hits with E-value less than e-5 from PSISearch2. For how to decide appropriate thresholds for C-value and E-value, please see the part ‘C-value to re-order hits close to E-value threshold’ in the Method section. The number of hits included by PSISearch2D is slightly larger than that by PSISearch2. More importantly, the number of true positive hits (165 690) by PSISearch2D is also larger than that (161 644) by PSISearch2 and the number of false positives (6740) by PSISearch2D is about 25% fewer than that (8873) by PSISearch2 (Table 1). The false-positive rates are 3.9% and 5.2% for PSISearch2D and PSISearch2, respectively. In summary, PSISearch2D finds out more true positive hits and at the same time results in fewer false positives compared with the original PSISearch2 tool. This indicates PSISearch2D outperforms PSISearch2 in terms of both accuracy and specificity.

**Table 1:** The summary result from the benchmarking between PSISearch2D and PSISearch2

	PSISearch2D			PSISearch2 [13]		
	Hits included	True positives	False positives	Hits included	True positives	False positives
Top 5 hits	368	330 (89.7%)	38 (10.3%)	368	311 (84.5%)	57 (15.5%)
Hits better than thresholds (C-value>6 or E-value<e-5)	172,430	165,690 (96.1%)	6740 (3.9%)	170,517	161,644 (94.8%)	8873 (5.2%)

### Evaluation in characterizing unknown proteins

Over 45 million protein sequences (more than 36% of proteins) in UniProtKB (Release 2018_08 12-Sep-2018) remain unknown or uncharacterized. With the support of domain annotation, PSISearch2D can be used to recharacterize these uncharacterized proteins. Unknown protein sequences, which contain descriptions of ‘unknown protein’ or ‘uncharacterized protein’ in protein description but with domain information, were retrieved from the UniProtKB database and defined as the testing query data set of unknown proteins. To evaluate PSISearch2D in characterizing unknown proteins, 139 503 unknown protein sequences of bacteria and 352 757 unknown protein sequences of virus were randomly selected from UniProtKB and searched against UniProtKB_SwissProt by the standalone version of PSISearch2D and the original PSISearch2 (13). The bacterial and viral data sets contain more uncharacterized protein data than eukaryotic sequences, therefore we chose the bacterial and viral data sets for testing. The results were compared to see how many unknown queries can be characterized by the two applications.

In a sequence homolog search, the information of the top hit (not the query itself) is usually used to characterize the query. In our test, if a query sequence has a top hit with clear description of the protein name, the query is defined as a characterized protein; if the top hit contains ambiguous descriptions, such as ‘unknown protein’, ‘uncharacterized protein’, but since any of the top five hits contain clear description of the protein name, matched domains to the query and qualified E-value, the query is still defined as characterized; otherwise, the query remains uncharacterized. For 139 503 unknown bacteria proteins, PSISearch2 provided annotation for 34 320 (24.6%) of them and PSISearch2D provided annotation for 77 695 (55.7%), about 2.3-fold more characterization than PSISearch2. For 352 757 unknown virus proteins, PSISearch2 provided annotation for 77 315 (21.9%) of them, and PSISearch2D provided annotation for 140 751 (39.9%), about 1.8-fold more characterization than PSISearch2.

It would be insufficient to use only the top hit to annotate queries; therefore, we conducted another similar test by using top five hits. In testing PSISearch2, the top five hits of each query with qualified E-value are included in the PSISearch2 hit set. In testing PSISearch2D, because matched domains provide support to the hits, the top five hits with clear description of protein name, matched domains to the query and qualified E-value are included in the hit set. For 139 503 queries of unknown bacteria proteins, 327 772 top 5 hits were retrieved in total. The 274 762 (83.8%) of the hits were provided with annotation by PSISearch2, and 301 779 (92.1%) were provided with annotation by PSISearch2D, about 9.8% more characterization than by PSISearch2. For 352 757 queries of unknown virus proteins, 481 908 top five hits were retrieved in total. The 409 513 (85.0%) of the hits were provided with annotation by PSISearch2, and 433 247 (89.9%) were provided with annotation by PSISearch2D, about 5.8% more characterization than by PSISearch2.

The UniProtKB Experimental Evidence describes the source of the information, e.g. an experiment that has been published in the scientific literature (https://www.uniprot.org/help/evidences). A matched protein with experimental evidence indicates that the search has resulted in a characterized protein. In order to classify how many hits are actually supported by experimental evidences, we extracted the evidence type information of Evidence and Conclusion Ontology (ECO) (26) for all proteins from the XML file in the current UniProt release and calculated the number of hits annotated with the experimental evidence (ECO:0000269). For the queries of unknown virus proteins, 124 457 of 481 908 top five hits (25.8%) by PSISearch2D have experimental evidences. For the queries of unknown bacteria proteins, 88 135 of 327 772 top five hits (26.9%) by PSISearch2D have experimental evidences. We further classified other 35.3% and 41.0% unknown virus and bacteria hits that have no experimental evidence but are supported by curator inference with manual assertion (ECO:0000305). This means that totally 61.1% and 67.9% top five PSISeach2D hits for unknown virus and bacteria proteins, respectively, are supported by annotation of either experimental evidences or manual curation inference in ECO.

### Case examples to characterize *E. coli* unknown proteins

**Figure 6:**
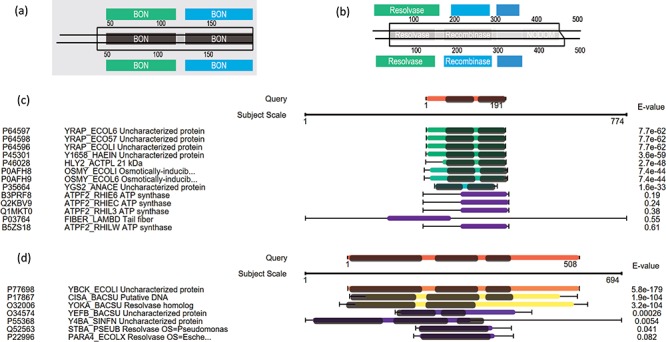
Case examples for *E. coli* uncharacterized proteins. (a) Protein domain visualization of YRAP_ECOLI and HLY2_ACTPL; (b) protein domain visualization of YBCK_ECOLI and CISA_BACSU; (c) (d) sequence and domain alignments are combinedly displayed in the view of multiple hits.

Here we use some case examples to demonstrate the use of PSISearch2D in characterizing unknown proteins. The 15 (~40%) of 40 uncharacterized *E. coli* proteins with domains were identified using PSI-Search2D against UniProtKB_SwissProt (Table 2). For example, the *E. coli* protein YraP (UniProtKB accession P64596) was originally marked as uncharacterized by the UniProt review team. By using PSISearch2D, the protein has a top hit HLY2_ACTPL 21 kDa hemolysin in *Actinobacillus pleuropneumoniae* and other two known hits in *E. coli* with good E-values. These proteins have two BON domains on the sequence alignment area (Figure 6a and c). In order to compare the annotations between PSISearch2D and InterProScan (14), we also analyzed the 15 proteins in Table 2 using InterProScan. The last three columns in Table 2 show the matched and mismatched domain annotations between the two applications. Twelve of the proteins have exactly the same or highly similar domain annotations from both applications, and only three of the proteins contain different domain annotations. InterProScan outputs the full names of domains instead of the short names. The difference might be due to the uses of difference domain databases in PSISearch2D and InterProScan. In Figure 6a, the top part shows the two BON domains in the query sequence; the bottom part indicates the same domains in the hit; in the middle part, the two lines represent the query and the hit sequences, respectively, the black-line box represents the sequence alignment between the query YraP and the subject HLY2_ACTPL, and the grey boxes inside the black-line box indicate that the matched domains of BON are aligned together along with the sequence alignment. Another example is the *E. coli* uncharacterized protein YbcK (UniProtKB Accession P77698), which matches the putative DNA recombinase in *Bacillus subtilis* by PSISearch2D. They both have three domains on the sequence alignment area (Figure 6b and d).

**Table 2:** Recharacterize the E. coli uncharacterized protein in UniProtKB_SwissProt

	Query		Top hit from PSISearch2D				Annotation from InterProScan	Same or highly similar domain annotation between the two applications
	ACC | ID	Description	ACC | ID	Protein name Description	Species	Matched domain(s) & regions	Matched domain(s)	
1	P64596 | YRAP_ECOLI	Uncharacterized protein YraP	P46028 | HLY2_ACTPL	21 kDa hemolysin	Actinobacillus pleuropneumoniae	BON1, BON2	BON, BON	Yes
2	P77698 | YBCK_ECOLI	Uncharacterized protein YbcK	P17867 | CISA_BACSU	Putative DNA recombinase	Bacillus subtilis	Resolvase, Recombinase, Zn_ribbon_recom	Resolvase, N-terminal catalytic, DNA-binding recombinase, Recombinase zinc beta ribbon	Yes
3	P18355 | YPFU_ECOLI	Uncharacterized protein in traD-traI intergenic region	O06663 | VAPB_SHIFL	Antitoxin VapB	Shigella flexneri	MazE_antitoxin	SpoVT-AbrB (Bacterial antibiotic resistance protein B)	No
4	P52096 | YAER_ECOLI	Uncharacterized protein YaeR	Q9KT93 | LGUL_VIBCH	Probable lactoylglutathione lyase	Vibrio cholerae serotype O1	Glyoxalase	Glyoxalase/fosfomycin resistance/dioxygenase, Vicinal oxygen chelate (VOC), YwkD-like	Yes
5	P65367 | YQCA_ECOLI	Uncharacterized protein YqcA	P37040 | NCPR_MOUSE	NADPH--cytochrome P450 reductase	*Mus musculus*	Flavodoxin_1	Flavodoxin/nitric oxide synthase	Yes
6	P0AFR4 | YCIO_ECOLI	Uncharacterized protein YciO	Q6LLJ9 | TSAC_PHOPR	Threonylcarbamoyl-AMP synthase	*Photobacterium profundum*	Sua5_yciO_yrdC (15-190)	YrdC-like	Yes
7	P37663 | YHJY_ECOLI	Uncharacterized protein YhjY	LIP1_PHOLU	Lipase 1	*Photorhabdus luminescens*	Autotransporter	Autotransporter beta	Yes
8	P64557 | YGFM_ECOLI	Uncharacterized protein YgfM	Q5FB27 | AOXA_MACFA	Aldehyde oxidase 1	*Macaca fascicularis*	FAD_binding_5, CO_deh_flav_C	FAD-binding domain, CO dehydrogenase flavoprotein (C-terminal), Molybdopterin dehydrogenase	Yes
9	P75863 | YCBX_ECOLI	Uncharacterized protein YcbX	Q96EN8 | MOCOS_HUMAN	Molybdenum cofactor sulfurase	*Homo sapiens,*	MOSC_N, MOSC	MOSC (N-terminal beta barrel), Molybdenum cofactor sulfurase (C-terminal), 2Fe-2S ferredoxin-type iron-sulfur binding	Yes
10	P52131 | YFJP_ECOLI	Uncharacterized protein YfjP	B9DNL3 | ERA_STACT	GTPase Era	*Staphylococcus carnosus*	MMR_HSR	GTP binding	No
11	Q47688 | YKFC_ECOLI	Putative uncharacterized protein YkfC	P0A3U0 | LTRA_LACLC	Group II intron-encoded protein LtrA	*Lactococcus lactis subsp. cremoris*	RVT_1	Reverse transcriptase	Yes
12	P31666 | YADE_ECOLI	Uncharacterized protein YadE	Q6G606 | ICAB_STAAS	Poly-beta-1,6-N-acetyl-D-glucosamine N-deacetylase	*Staphylococcus aureus*	Polysacc_deac_1	NodB homology	No
13	P77286 | YDEU_ECOLI	Uncharacterized protein YdeU	Q7BCK4 | ICSA_SHIFL	Outer membrane protein IcsA autotransporter	*Shigella flexneri*	Autotransporter	Autotransporte_beta	Yes
14	Q46820 | YGFT_ECOLI	Uncharacterized protein YgfT	P37127 | AEGA_ECOLI	Protein AegA	*Escherichia coli* (strain K12)	Fer4, Fer4_20 FAD_binding_3, Pyr_redox_2, NAD_binding_8	4Fe-4S ferredoxin-type, iron-sulphur binding; Dihydroprymidine dehydrogenase domain II; FAD/NAD(P)-binding	Yes
15	P76347 | YEEJ_ECOLI	Uncharacterized protein YeeJ	P11922 | INVA_YERPS	Invasin	*Yersinia pseudotuberculosis* serotype I	IAT_beta, Invasin_D3, Big_1	Inverse autotransporter, beta; Invasin, domain 3; Big-1 (bacterial Ig-like domain 1)	Yes

### Other new features and availability

PSISearch2D provides advanced new features such as GGSEARCH-/GLSEARCH-/PSI-BLAST-based iterative search as extra options for global-global, global-local and psi-blast sequence alignments, respectively; the new auto-iteration mode allows multiple iterative searches that are greatly needed in high-throughput protein functional prediction and genome annotation. Its web application achieves much better selectivity compared with other iterative protein search servers at a similar speed. It completes a 10-iteration search against UniProtKB_SwissProt in about 20 s, over 4-fold faster than the previous version and at a similar speed of PSI-BLAST and jackHMMER.

The PSISearch2D web application can be found from the official website http://lilab2.sysu.edu.cn/psisearch2d. Its standalone program, source codes, web services API client programs and relevant domain databases are also freely available from the website. All supporting materials, including the full documentation and the testing data sets for input and output, can be freely downloaded from the website.

## Discussion and conclusion

Iterative homolog search plays an important role in detection of remotely related proteins. Most of the current tools of iterative sequence search result in HOE errors (11). More sensitive scoring models and better alignment visualization are highly needed to improve the predictive performance for protein remote homology detection (1). Therefore, we develop the PSISearch2D tool with significantly new features including the combined visualization of sequence and domain alignments and the newly defined C-value.

PSISearch2D offers three major advantages. First, the combined visualization of sequence and domain alignments improves the result interpretation for resulting hits. Other tools also offer different styles of domain visualizations. For example, the domain annotation in SUPERFAMILY (27) is based on a collection of Hidden Markov Models, which represent structural protein domains at the SCOP (28) superfamily level. The SUPPERFAMILY web tool provides domain features in visualization but does not show combined alignments of sequence and domains. The HMMER web application is another example for predicting sequence features including domain architectures of matches. PSISearch2D produces matches through iterative sequence search, reorders matches with the newly define score C-value and demonstrates both sequence and domain alignments in novel visualizations. Compared with currently available web applications, PSISearch2D allows comparisons between the two types alignments to find agreements or disagreements between them in order to support better result curation and provide more clues for further analysis. PSISearch2D improves the characterization of unknown proteins but is not supposed to replace the current methods for protein domain prediction. Second, it helps to identify mismatched hits with E-values better than or close to thresholds. The re-ordering of hits by the newly defined C-value can filter out most of the mismatches close to E-value thresholds. Also, false-positive hits with good E-values can be excluded with the support of combined alignment visualization in the view of multiple hits. Third, PSISearch2D can characterize unknown proteins with the information from confidently supported domains. For example, PSISearch2D has provided annotation for 77 695 of 139 503 unknown bacteria proteins and 140 751 of 352 757 unknown virus proteins in UniProtKB, about 2.3-fold and 1.8-fold more characterization than PSISearch2 (13), respectively. All of these advantages benefit the remotely homologous protein searches.

C-value is designed to assess the hits that have E-values close to the chosen threshold, while E-value is sufficient to assess the hits that have E-values much less than the threshold. In some of the cases, the alignment of matched domains can give more confidence to interpret the hits. The domain annotation and visualization cost more computing resources, ~3% slower than PSISearch2 (13) in speed. However, with this small cost in speed, PSISearch2D results in much better interpretation of hits and more characterized features of query proteins.

In PSISearch2D, domain alignments are used to assess and interpret the resulting hits. In the future, we plan to include the information from domain alignments into the phase of PSSM construction (10). This might be able to directly and greatly improve the score model of iterative searches, and thus characterize more remotely homologous proteins as well as provide more confident matches at the same time.

In conclusion, PSISearch2D enhances remotely related protein search and potentially improves protein functional prediction and genomic annotation. Its novel visualization of combined sequence and domain alignments ease and enable better result interpretation. PSISearch2D can be used to improve the characterization of unknown proteins by search remotely related protein data sets. Additional alignment methods including GGSEARCH, GLSEARCH and PSI-BLAST provide choices for optional global and local sequence iterative searches. Auto-iteration mode allows multiple iterative searches and thus greatly facilitates high-throughput protein functional prediction and genome annotation.

## Notes

**Database URL**: http://lilab2.sysu.edu.cn/psisearch2d
